# Refining the link between REM sleep behavior disorder and neurodegeneration: Genetic correlation, Mendelian randomization, and colocalization evidence

**DOI:** 10.1097/MD.0000000000048922

**Published:** 2026-07-10

**Authors:** Hong Ye, Jia-Li Wang, Qiu-Han Xu, Yu-Liang Gao

**Affiliations:** a Department of Neurology, Xiangshan Hospital of TCM Medical and Health Group, Ningbo City, Zhejiang Province, China; b Department of Neurology, Second Affiliated Hospital, College of Medicine, Zhejiang University, Hangzhou, Zhejiang, China; c Department of Neurosurgery, First Affiliated Hospital, College of Medicine, Zhejiang University, Hangzhou, Zhejiang, China; d Department of Neurology, Quzhou Hospital of Traditional Chinese Medicine, Quzhou, Zhejiang, China.

**Keywords:** causal relationship, colocalization analysis, isolated REM sleep behavior disorder, linkage disequilibrium score regression, Mendelian randomization, neurodegenerative diseases

## Abstract

Observational studies have proposed a link between isolated rapid eye movement sleep behavior disorder (iRBD) and several neurodegenerative diseases. We employed genome-wide linkage disequilibrium score regression (LDSC), standard two-sample Mendelian randomization (MR), and colocalization analysis to assess the causal links between iRBD and these neurodegenerative conditions. iRBD demonstrated a positive causal association with Alzheimer disease (odds ratio [OR] = 1.02, 95% confidence interval [CI]: 1.00–1.03, *P* = 1.10E−02), Parkinson disease (OR = 1.10, 95% CI: 1.03–1.16, *P* = 2.96E−03), and multiple sclerosis (OR = 1.09, 95% CI: 1.02–1.17, *P* = 1.61E−02). A strong positive genetic correlation with dementia with Lewy bodies was observed (rg = 1.6313, *P* = .0002), along with a causal association (OR = 1.45, 95% CI: 1.03–2.06, *P* = 3.53E−02), further supported by colocalization analysis. No significant causal relationship was identified between iRBD and amyotrophic lateral sclerosis (all *P* > .05). Additionally, reverse Mendelian randomization analyses did not reveal any causal relationships between the neurodegenerative diseases studied and iRBD. Our findings provide robust genetic evidence supporting a causal relationship between iRBD and the risk of multiple neurodegenerative diseases, highlighting the potential for shared pathophysiological mechanisms.

## 1. Introduction

The aging global population presents humanity with unprecedented challenges, particularly the rising burden of neurodegenerative diseases.^[1–3]^ These conditions, characterized by progressive neuronal loss and the deterioration of neural networks, severely impact millions of lives worldwide, impairing memory, cognition, behavior, and motor function.^[4]^ Neurodegenerative diseases often manifest insidiously and worsen irreversibly over time, predominantly affecting older adults.^[5]^ Despite their growing prevalence, therapeutic options remain limited, and early detection strategies are scarce.^[6,7]^ Therefore, identifying modifiable risk factors is critical for advancing prevention and improving patient outcomes.

Isolated rapid eye movement (REM) sleep behavior disorder (iRBD) is a parasomnia characterized by the loss of normal muscle atonia during REM sleep, leading to dream enactment behaviors, often accompanied by vocalization.^[8]^ This disruption significantly impacts sleep quality and poses safety risks for both patients and their bed partners. Notably, iRBD is widely recognized as a precursor to neurodegenerative α-synucleinopathies, including Parkinson disease (PD) and dementia with Lewy bodies (DLB), with studies indicating that over 80% of iRBD patients progress to PD or DLB within approximately 14 years.^[9,10]^ However, emerging evidence suggests that iRBD is not exclusive to synucleinopathies and may also be linked to other neurodegenerative diseases, such as Alzheimer disease (AD), amyotrophic lateral sclerosis (ALS), and MS.^[11,12]^

Despite the observed associations between iRBD and neurodegenerative diseases, most evidence remains observational, leaving their causal relationships largely unexplored. To address this gap, it is essential to investigate the causal effects of iRBD on neurodegenerative diseases using a MR approach.

**Figure 1.**
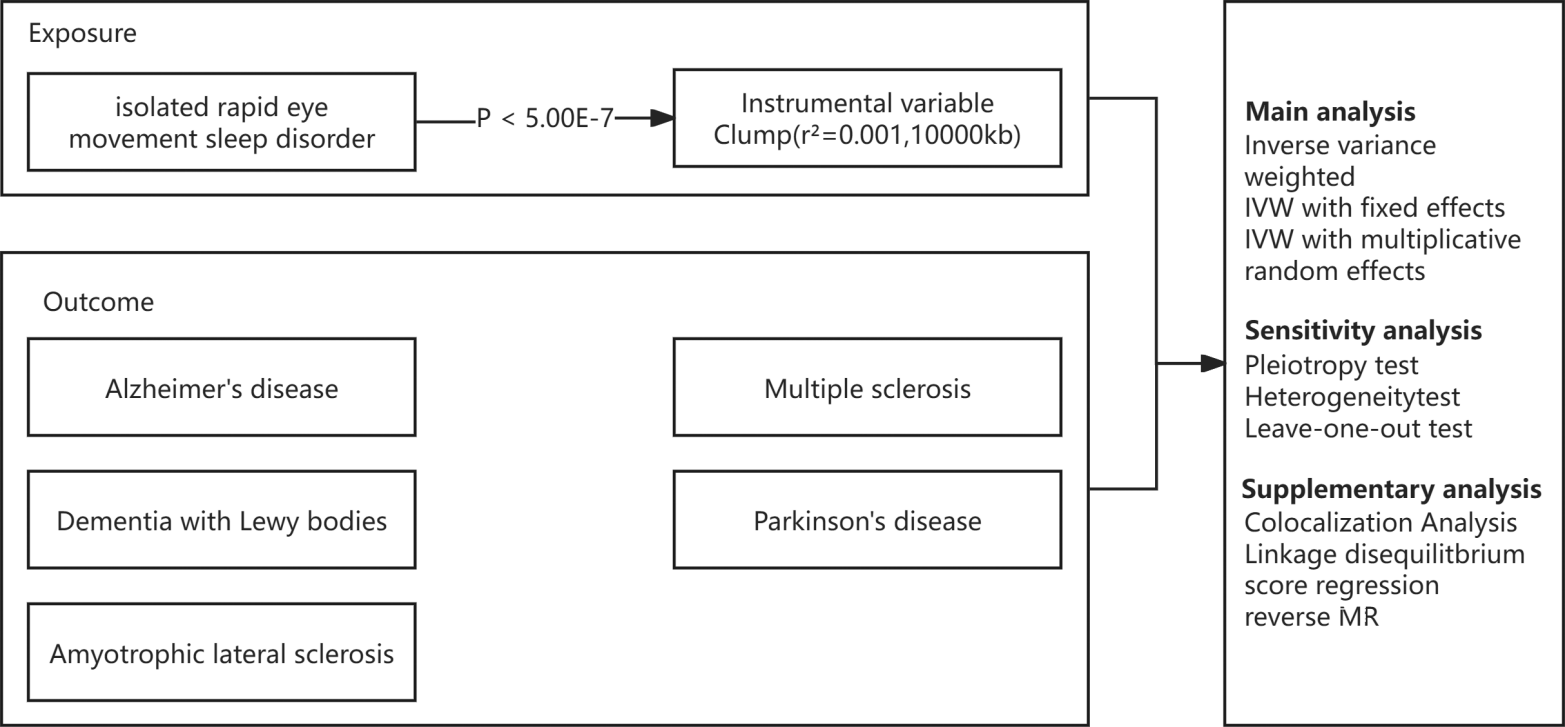
Flowchart of the study.

In this study, we leveraged genome-wide linkage disequilibrium score regression (LDSC), standard two-sample MR, and colocalization analyses, integrating large-scale genome-wide association study (GWAS) data to assess the potential causal relationships between iRBD and neurodegenerative diseases, with a focus on AD, PD, DLB, MS, and ALS. The study workflow is depicted in Figure 1, and the research strictly adheres to the Strengthening the Reporting of Observational Studies in Epidemiology using Mendelian randomization guidelines,^[13]^ ensuring methodological rigor and transparency.

## 2. Methods

### 2.1. Study design and data sources

This study used a two-sample MR design to assess the causal relationship between iRBD and the risk of neurodegenerative diseases, including PD, AD, ALS, MS, and DLB. We utilized summary-level GWAS data for both iRBD (exposure) and the neurodegenerative outcomes (PD, AD, ALS, MS, and DLB). The GWAS data for iRBD were sourced from the GWAS Catalog (GCST90204200),^[14]^ comprising 1061 cases and 8386 controls, with over 7.7 million single nucleotide polymorphisms (SNPs). Similarly, large-scale GWAS datasets were used for the neurodegenerative diseases, each with significant sample sizes and comprehensive SNP data. Detailed information on the data sources, sample sizes, and SNPs for each trait is provided in Table 1.

**Table 1:** Summary of GWAS Data for iRBD and associated neurodegenerative diseases.

Trait	Data source	Sample size	SNPs in GWAS	Year	Description
Exposure					
iRBD	GWAS catalog: GCST90204200	9447	11,735,271	2022	The iRBD cohort (*N* cases = 1061, *N* controls = 8386) included large groups from French, Canadian, Italian, and British origins, along with smaller cohorts from various European populations.^[14]^
Outcome					
AD	ebi-a-GCST005921	314,278	7,746,640	2018	The GWAS data for Alzheimer disease with a family history included 42,034 AD patients and 272,244 controls, and was used as summary association statistics for exposure.^[15]^
MS	ieu-b-18	115,803	6,304,359	2019	The multiple sclerosis dataset from the International Multiple Sclerosis Genetics Consortium includes 47,429 cases, 68,374 controls, and 6,304,359 SNPs from a European population.^[16]^
DLB	ebi-a-GCST90001390	6618	7,593,175	2021	The dementia with Lewy bodies dataset, authored by Chia R, includes 2591 cases, 4027 controls, and 7,593,175 SNPs from a European population.^[17]^
PD	ieu-b-7	482,730	17,891,936	2019	The Parkinson disease dataset, part of the International Parkinson Disease. Genomics Consortium and authored by Nalls MA, consists of 33,674 cases, 449,056 controls, and 17,891,936 SNPs from a European population.^[18]^
ALS	ebi-a-GCST90013429	84,694	10,181,076	2020	The amyotrophic lateral sclerosis dataset, authored by Iacoangeli A, includes 22,040 cases, 62,644 controls, and 10,181,076 SNPs from a mixed population.^[19]^

Comprehensive information is provided in the table, detailing the traits, data sources, sample sizes, and SNP counts for each dataset. The table outlines the key attributes and origins of each dataset.

AD = Alzheimer disease, ALS = amyotrophic lateral sclerosis, DLB = dementia with Lewy bodies, EBI = European Bioinformatics Institute, GWAS = genome-wide association study, IEU = integrative epidemiology unit, iRBD = isolated REM sleep behavior disorder, MS = multiple sclerosis, PD = Parkinson disease, SNP = single nucleotide polymorphism.

### 2.2. Selection of genetic instruments

Genetic instruments for iRBD were selected based on SNPs that reached a relaxed genome-wide significance threshold of *P* < 5.00E−7 in the GWAS for iRBD. This threshold was chosen to increase the number of available SNPs for analysis, as a more stringent threshold (*P* < 5.00E−8) resulted in too few SNPs to be effective for robust analysis. To ensure independence between SNPs, linkage disequilibrium (LD) clumping was applied using an *r*^2^ threshold of 0.001 and a clumping window of 10,000 kb. Palindromic or ambiguous SNPs were excluded from the analysis to avoid strand orientation issues, and proxy SNPs were utilized where necessary to maintain instrument strength.

### 2.3. Statistical analysis

We conducted MR analyses using the TwoSampleMR package in R version 4.4.0. The primary method for estimating causal effects was the inverse-variance weighted (IVW) method. IVW with fixed effects (IVW-FE) was used when there were fewer than 3 valid SNPs, while IVW with multiplicative random effects (IVW-MRE) was applied to account for heterogeneity when detected, provided the number of valid SNPs exceeded 3. To ensure robustness, we applied 2 additional methods: weighted median and weighted mode. Sensitivity analyses included Cochran *Q* test to assess heterogeneity and the MR-Egger intercept to evaluate horizontal pleiotropy. If heterogeneity was present, IVW-MRE was used. Leave-one-out analysis was applied to identify influential SNP outliers, and funnel plots were generated to visualize SNP heterogeneity. Forest and scatter plots were created to illustrate the results. Additionally, a reverse MR analysis was conducted to assess the possibility of reverse causality.

### 2.4. Genetic correlation analysis

LDSC^[20]^ was performed to estimate the genetic correlation (rg) between iRBD and each of the neurodegenerative diseases. LDSC assesses the extent of shared genetic architecture between the traits by examining the correlation in effect sizes of SNPs across the GWAS datasets. Significant genetic correlation (*P* < .05) indicates a shared genetic basis between iRBD and the neurodegenerative disease.

### 2.5. Colocalization analysis

Colocalization analysis was conducted using the colocalization analysis package package to determine whether the same causal variants underlie both iRBD and each neurodegenerative disease. The colocalization analysis package analysis calculates posterior probabilities for 5 hypotheses (H0–H4) to assess the likelihood of shared causal variants.^[21]^ A posterior probability (PP.H4) >0.8 was considered strong evidence of colocalization, suggesting that the same genetic variants influence both iRBD and the neurodegenerative disease.

## 3. Results

Following the clustering process, LD-independent SNPs for iRBD were identified. To ensure robust instrument selection, the following criteria were applied to exclude specific SNPs: if a given SNP was not present in the outcome datasets (AD, DLB, PD, MS, ALS) and no proxy in LD could be identified; if the SNP was ambiguous or palindromic with ambiguous strand orientation, rendering correction impossible. Consequently, SNPs retained as instrumental variables (IVs) for further analysis are listed in Table S1. The *F*-statistic for each IV-exposure association exceeded 10, indicating that weak instrument bias is unlikely in our study.

**Figure 2.**
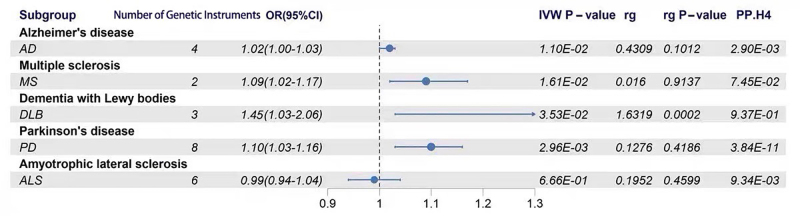
Causal associations and genetic correlations between iRBD and neurodegenerative diseases. Odds ratios (OR) with 95% confidence intervals (CI) are shown for AD, MS, DLB, PD, and ALS based on Mendelian randomization analysis (IVW *P* values). Genetic correlations (r_9_) and colocalization probabilities (PP.H4) are also presented, highlighting significant links, particularly with DLB and PD. AD = Alzheimer disease, ALS = amyotrophic lateral sclerosis, DLB = dementia with Lewy bodies, iRBD = isolated REM sleep behavior disorder, IVW = inverse-variance weighted, MS = multiple sclerosis, PD = Parkinson disease, PP.H4 = posterior probability of hypothesis 4 (shared causal variant).

We investigated the causal impact of iRBD on 5 major neurodegenerative diseases: AD, DLB, PD, MS, and ALS (Fig. 2). In this MR study, we investigated the association between iRBD and 5 neurodegenerative diseases. For AD (ebi-a-GCST005921), 4 SNPs were included as IVs. As no heterogeneity was detected, the IVW fixed-effects (IVW-FE) model was considered the primary analysis. The IVW-FE method demonstrated a significant association (OR [95% confidence interval (CI)] = 1.02 [1.00–1.03], *P* = .011). The weighted median approach yielded a consistent result (*P* = .031), whereas the weighted mode method did not reach statistical significance. The IVW multiplicative random-effects (IVW-MRE) model produced a similar effect estimate; minor differences in *P*-values between IVW-FE and IVW-MRE reflect differences in variance estimation under the 2 modeling assumptions rather than substantive discrepancies in effect size. No heterogeneity (*P* = .999) or pleiotropy (*P* = .947) was observed. For MS (ieu-b-18), with 2 SNPs, we used the IVW-FE method due to the small SNP number, and a significant association was found (OR [95% CI] = 1.088 [1.015–1.165], *P* = .016). Heterogeneity was non-significant (*P* = .969), and pleiotropy could not be assessed. For DLB (ebi-a-GCST90001390), with 3 SNPs, the IVW-MRE method showed a significant association (OR [95% CI] = 1.45 [1.03–2.06], *P* = .035). The IVW-FE method showed *P* = 1.53E−7, weighted median *P* = .008, and weighted mode *P* = .042. No pleiotropy was observed (*P* = .413964), and while heterogeneity was present (*P* = .001991), the IVW-MRE result supports the robustness of the association. For PD (ieu-b-7), with 8 SNPs, the IVW-MRE method showed a significant association (OR [95% CI] = 1.096 [1.031–1.164], *P* = .0029602). The IVW-FE method *P* = .0025265, weighted median *P* = .006948, and weighted mode *P* = .0208873 results were consistent, supporting the robustness of the association. No heterogeneity (*P* = .4053897) or pleiotropy (*P* = .2389114) was observed. For ALS (ebi-a-GCST90013429), with 6 SNPs, the IVW method did not show a significant association (OR [95% CI] = 0.99 [0.95–1.03], *P* = .666), indicating no association between iRBD and ALS.

The sensitivity analyses confirmed the robustness of the MR estimates. Despite the IVW methods being susceptible to weak IV bias, the weighted median and weighted mode methods were consistent with the MR estimates. Heterogeneity was evaluated using Cochran *Q* statistic, and pleiotropy was assessed with the MR-Egger intercept (Table 2). The leave-one-out sensitivity analysis and forest plots (Fig. S1) did not reveal any anomalous SNPs, confirming the stability of the MR estimates. Scatter plots and funnel plots (Fig. S1) further supported these findings, showing directions consistent with the IVW approach and symmetrical distribution of IVs. Additional reverse MR analyses for the diseases showed no significant associations: for DLB, the IVW method yielded *P* = .0913; for ALS, *P* = .2441; for AD, *P* = .2298; and for PD, *P* = .0691. Reverse MR for MS was not performed due to insufficient SNPs (Table S2).

**Table 2:** Sensitivity analysis of Mendelian randomization estimates: robustness, heterogeneity, and pleiotropy assessment.

Exposure	*R* ^2^	*F* statistics median	MR-Egger		Cochran *Q*		MR-Steiger	
			Intercept	*P*	*Q*	*P*	Correct causal direction	*P*
AD	0.004	36.96	−0.001	.947	0.020	.999	TRUE	4.33e−31
MS	0.005	47.97	NA	NA	0.001	.969	TRUE	1.70e−18
DLB	0.004	40.74	1.093	.413	12.438	.002	TRUE	2.35e−02
PD	0.003	32.37	0.050	.239	7.229	.405	TRUE	2.39e−46
ALS	0.004	33.67	−0.047	.430	6.718	.242	TRUE	4.10e−37

AD = Alzheimer disease, ALS = amyotrophic lateral sclerosis, DLB = dementia with Lewy bodies, MS = multiple sclerosis, PD = Parkinson disease.

As illustrated in Figure 2, the genetic correlation between iRBD and these neurodegenerative disorders spans from 0.016 to 1.6319. A significant genetic correlation was observed between iRBD and DLB (*P* = .0002), while no notable genetic correlations were found with the other diseases. Colocalization analysis further supports the presence of a shared genetic basis between iRBD and DLB (PP.H4 = 9.37E−01). Conversely, weak colocalization signals were observed for PD, AD, MS, and ALS, as detailed in Table S3 and Figure S2.

## 4. Discussion

Leveraging a large-scale GWAS dataset, we investigated the causal relationship between iRBD and multiple neurodegenerative diseases using MR and colocalization analyses. Our study provides robust genetic evidence linking iRBD to AD, PD, DLB, and MS. These findings underscore the intricate relationship between sleep disturbances and neurodegeneration, offering critical insights into the potential for early detection and prevention of these disorders.

### 4.1. Alzheimer disease association

A central finding of our study is the identification of a positive causal association between iRBD and AD (OR = 1.02, 95% CI: 1.00–1.03, *P* = 1.10 × 10^−2^). This observation suggests a potential shared pathological mechanism linking iRBD and AD. While iRBD is predominantly recognized as a robust predictor of neurodegeneration in synucleinopathies, accumulating evidence also indicates a frequent coexistence of iRBD and AD in clinical settings.^[12]^ Previous studies report that 24.5 to 40% of AD patients exhibit sleep disturbances, primarily characterized by frequent nocturnal awakenings, nighttime restlessness, and excessive daytime somnolence.^[22]^ In a longitudinal study with a 4.2-year follow-up, 18 out of 84 iRBD patients developed neurodegenerative disorders, with 3 diagnosed with AD.^[23]^ Similarly, in another cohort of 179 iRBD patients followed for an average of 5.8 years, 50 (27.9%) progressed to neurodegenerative diseases, and 14 were diagnosed as probable AD.^[24]^ These findings underscore the necessity for further investigation into the intersection of iRBD and AD pathophysiology.

The precise biological mechanisms linking iRBD and AD remain to be fully elucidated. Current models of sleep-dependent memory consolidation suggest that REM sleep plays a critical role in the pruning of unstable dendritic spines formed during non-rapid eye movement sleep and the stabilization of those involved in memory formation.^[25–27]^ As such, degeneration of cholinergic nuclei in the brainstem and basal forebrain, regions crucial for REM sleep production, may impair these sleep-dependent memory processes.^[28,29]^ Furthermore, acetylcholine, a neurotransmitter pivotal for inducing muscle atonia during REM sleep, is implicated in the shared physiological mechanisms underlying both iRBD and AD. Reduced acetylcholine levels, along with noradrenergic imbalances driven by locus coeruleus atrophy, are thought to be key contributors to the pathophysiology of iRBD in AD.^[30]^ Additionally, sleep disturbances associated with iRBD may exacerbate amyloid-beta and tau pathologies, further accelerating cognitive decline.^[31]^

Our findings provide additional genetic evidence supporting the association between iRBD and AD, indicating that iRBD may serve as an early neurodegenerative marker not only for synucleinopathies but also for amyloid-beta and tauopathies. These insights could pave the way for early therapeutic interventions, with sleep-based treatments potentially delaying the onset or progression of cognitive decline.

### 4.2. Parkinson disease and dementia with Lewy bodies association

Our analysis strengthens the findings of previous large observational studies, providing a higher level of clinical evidence. iRBD demonstrated a strong causal relationship with both PD (OR = 1.10, 95% CI: 1.03–1.16, *P* = 2.96 × 10^−3^) and DLB (OR = 1.45, 95% CI: 1.03–2.06, *P* = 3.53 × 10^−2^). Colocalization analysis further reinforced these associations, particularly in DLB, where a significant genetic overlap between iRBD and DLB was identified (rg = 1.6313, *P* = .0002). iRBD is a core clinical criterion for DLB diagnosis and serves as a prodromal biomarker in studies of prodromal PD.^[8,32]^ The high genetic correlation observed in our study highlights a shared pathophysiological mechanism between iRBD and DLB, while also defining the genetic relationship between iRBD, PD, and DLB.

Notably, significant heterogeneity was observed in the DLB analysis (Cochran *Q P* = .002). Although this was addressed using the IVW multiplicative random-effects model, which accounts for between-variant variability, several factors may contribute to this finding. Biologically, DLB is a heterogeneous disorder often characterized by mixed neuropathology, including α-synuclein, amyloid-β, and tau pathology, which may lead to variability in SNP-specific effects. Methodologically, the relatively modest sample size of the DLB GWAS and potential differences in case ascertainment across cohorts may also contribute to heterogeneity. Nevertheless, the consistent direction of effect and the robustness of the IVW-MRE results support the overall stability of the association.

### 4.3. Multiple sclerosis association

Our study revealed a significant association between iRBD and MS (OR = 1.09, 95% CI: 1.02–1.17, *P* = 1.61 × 10^−2^), broadening the spectrum of neurodegenerative diseases linked to iRBD and suggesting a potential role for REM sleep disturbances in the pathogenesis of demyelinating diseases. Sleep disturbances are prevalent among MS patients, with nearly half experiencing impaired sleep, which can exacerbate core symptoms such as fatigue, cognitive decline, and mood disorders.^[33,34]^ Clinical studies have shown that poor sleep quality in MS patients is associated with higher relapse rates.^[35]^ The specific mechanisms by which sleep disorders like iRBD influence MS onset and relapse remain unclear. One hypothesis is that iRBD may promote systemic inflammation, as indicated by elevated levels of proinflammatory cytokines and C-reactive protein, which could, in turn, accelerate neuronal damage and demyelination in MS.^[36,37]^ Moreover, numerous studies have demonstrated the essential role of sleep in neuroprotection and neural repair, suggesting that sleep disturbances may impair the brain’s capacity to recover from MS-related damage.^[38]^ The positive causal relationship between iRBD and MS identified in our study suggests that sleep disorders may serve as early warning signs of MS, particularly in individuals exhibiting abnormal sleep behavior. Further research is warranted to explore this novel association and to determine whether targeting REM sleep disturbances could reduce the incidence or progression of MS.

### 4.4. Advantages and limitations

Our study offers several important strengths. By integrating genetic correlation, MR, and colocalization analyses, we leveraged the complementary strengths of these approaches to address potential false-negative and false-positive results. This work represents a milestone as the first to explore the epidemiological relationship between iRBD and neurodegenerative diseases through genetics-driven evidence. The genetically-informed estimates provided in this study offer a more objective and precise assessment of the associations between iRBD and neurodegenerative diseases, mitigating biases commonly found in observational studies.

However, several limitations merit careful consideration. First, although statistically significant associations were identified for AD and MS, the observed effect sizes were modest. These estimates reflect relatively small increases in genetically predicted risk at the individual level and should not be interpreted as large clinical risk increments. While even modest genetic effects may have potential relevance at the population level – particularly for common, age-related neurodegenerative diseases – the clinical implications of these findings remain limited and require cautious interpretation. Second, although all IVs demonstrated adequate strength (*F*-statistics > 10), thereby minimizing the likelihood of weak instrument bias, the number of genome-wide significant SNPs available for MS (2 SNPs) was limited. A restricted number of instruments may increase the variance of causal estimates, reduce precision, and limit the robustness of sensitivity analyses for horizontal pleiotropy. Therefore, the causal estimate for MS should be interpreted cautiously, as its stability may be affected by the small number of genetic variants. Replication in larger GWAS with more comprehensive IVs will be essential to confirm this association. In addition, our analysis was restricted to individuals of European ancestry, which may limit the generalizability of these findings to other populations. The use of GWAS summary statistics also precluded stratified analyses by age, sex, or disease stage. Finally, causal relationships between iRBD and other neurodegenerative diseases could not be evaluated due to the lack of sufficiently powered summary datasets. Future studies incorporating larger, multi-ethnic cohorts and expanded genetic instruments will be necessary to validate and extend our findings.

## 5. Conclusion

In conclusion, our findings provide robust genetic evidence for the causal role of iRBD in multiple neurodegenerative diseases, including AD, PD, DLB, and MS. These results underscore the importance of sleep regulation in the early stages of neurodegeneration and highlight iRBD as a key factor in understanding the etiology and progression of these diseases. Further research into the molecular underpinnings of these associations could pave the way for novel diagnostic and therapeutic strategies aimed at preserving REM sleep and mitigating neurodegenerative disease risk.

## Acknowledgments

We acknowledge the contributions of the Genome-Wide Association Study database.

## Author contributions

**Conceptualization:** Hong Ye, Jia-Li Wang.

**Data curation:** Hong Ye, Jia-Li Wang.

**Formal analysis:** Hong Ye.

**Funding acquisition:** Hong Ye, Qiuhan Xu, Yu-Liang Gao.

**Investigation:** Jia-Li Wang.

**Methodology:** Jia-Li Wang.

**Project administration:** Qiuhan Xu, Yu-Liang Gao.

**Resources:** Jia-Li Wang.

**Software:** Qiuhan Xu.

**Supervision:** Qiuhan Xu.

**Validation:** Jia-Li Wang.

**Visualization:** Jia-Li Wang.

**Writing – original draft:** Hong Ye, Jia-Li Wang.

**Writing – review & editing:** Qiuhan Xu, Yu-Liang Gao.










