# Supplementary figures and images for: Refining the link between REM sleep behavior disorder and neurodegeneration: Genetic correlation, Mendelian randomization, and colocalization evidence
Source: Medicine (Baltimore). 2026 Jul 10;105(28):e48922. doi: 10.1097/MD.0000000000048922 (PMC13362855; doi:10.1097/MD.0000000000048922)

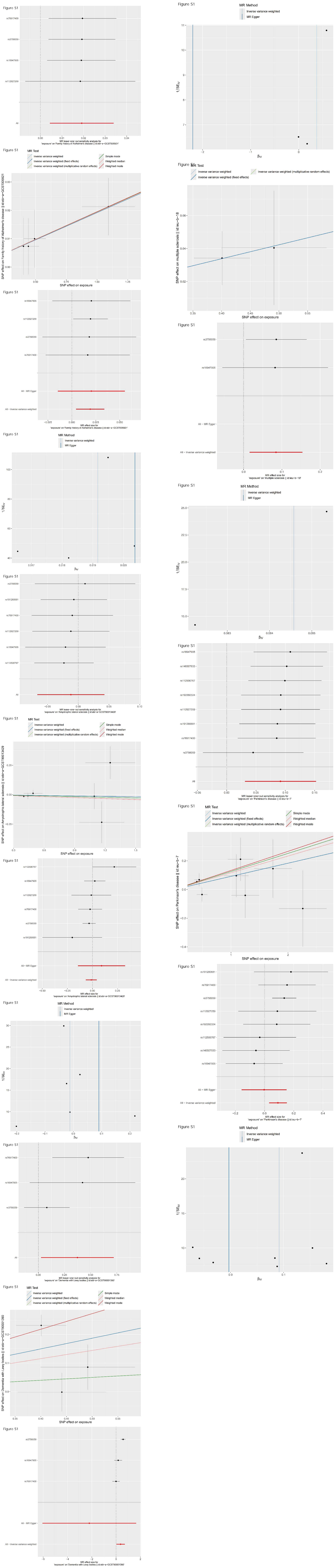

Supplement: Supplementary file 2 [file medi-105-e48922-s002.tiff]
